# Metformin maintains intrahepatic triglyceride content through increased hepatic *de novo* lipogenesis

**DOI:** 10.1530/EJE-21-0850

**Published:** 2022-01-17

**Authors:** Charlotte J Green, Thomas Marjot, John Walsby-Tickle, Catriona Charlton, Thomas Cornfield, Felix Westcott, Katherine E Pinnick, Ahmad Moolla, Jonathan M Hazlehurst, James McCullagh, Jeremy W Tomlinson, Leanne Hodson

**Affiliations:** 1 Oxford Centre for Diabetes, Endocrinology and Metabolism, University of Oxford, Churchill Hospital, Oxford, UK; 2 Translational Gastroenterology Unit, NIHR Oxford Biomedical Research Centre, University of Oxford, John Radcliffe Hospital, Oxford, UK; 3 Chemistry Research Laboratory, University of Oxford, Oxford, UK; 4 Institute of Metabolism and Systems Research, University of Birmingham, Edgbaston, Birmingham, UK; 5 NIHR Oxford Biomedical Research Centre, Oxford University Hospital Trusts, Oxford, UK

## Abstract

**Objective:**

Metformin is a first-line pharmacotherapy in the treatment of type 2 diabetes, a condition closely associated with non-alcoholic fatty liver disease (NAFLD). Although metformin promotes weight loss and improves insulin sensitivity, its effect on intrahepatic triglyceride (IHTG) remains unclear. We investigated the effect of metformin on IHTG, hepatic *de novo* lipogenesis (DNL), and fatty acid (FA) oxidation *in vivo* in humans.

**Design and methods:**

Metabolic investigations, using stable-isotope tracers, were performed in ten insulin-resistant, overweight/obese human participants with NAFLD who were treatment naïve before and after 12 weeks of metformin treatment. The effect of metformin on markers of s.c. adipose tissue FA metabolism and function, along with the plasma metabolome, was investigated.

**Results:**

Twelve weeks of treatment with metformin resulted in a significant reduction in body weight and improved insulin sensitivity, but IHTG content and FA oxidation remained unchanged. Metformin treatment was associated with a significant decrease in VLDL-triglyceride (TG) concentrations and a significant increase in the relative contribution of DNL-derived FAs to VLDL-TG. There were subtle and relatively few changes in s.c. adipose tissue FA metabolism and the plasma metabolome with metformin treatment.

**Conclusions:**

We demonstrate the mechanisms of action of metformin whereby it improves insulin sensitivity and promotes weight loss, without improvement in IHTG; these observations are partly explained through increased hepatic DNL and a lack of change in FA oxidation.

## Introduction

Non-alcoholic fatty liver disease (NAFLD) (also known as metabolic associated fatty liver disease (MAFLD) (1)) affects approximately 25% of the global population (2). NAFLD is strongly associated with obesity, type 2 diabetes (T2D), and cardiovascular disease (CVD) and begins with pathological accumulation of intrahepatic triglyceride (IHTG) (steatosis) which can progress to more severe liver disease (1). The accumulation of IHTG represents an imbalance between the amount of fatty acids (FA) entering the liver, FA synthesis (*de novo* lipogenesis (DNL)) within the liver, and FA disposal (via oxidation or export as triglyceride (TG) in VLDL) from the liver (3); insulin plays a key role in the regulation of these processes (3). Enhanced hepatic DNL has been suggested to contribute towards insulin resistance and NAFLD (4). Insulin resistance is often considered a hallmark of NAFLD and is the best predictor of liver disease progression (5).

In patients with T2D, it is well recognised that treatment with metformin leads to improvements in glycaemic control and insulin sensitivity. Based on observations in rodent models, metformin has been suggested as a possible candidate for the pharmacological treatment of NAFLD (6), an area of significant unmet clinical need as there is currently no licenced drug therapy, with the treatment and management of NAFLD being focussed predominantly on lifestyle and dietary modification (7). This contrasts with the multiple licenced medications available for the treatment of T2D. However, data are conflicting with the positive impact of metformin on IHTG being observed in rodent models not reliably translating into human studies (8). While rodent models have shown metformin to reduce IHTG through AMP-activated protein kinase (AMPK)-mediated FA oxidation and decreased hepatic DNL (8), meta-analyses of human trials suggest no histological benefit despite improvements in body weight and glycaemic control (9, 10). We therefore aimed to explain this disconnect by using experimental medicine and stable-isotope tracer techniques in an *in vivo* human study to characterise FA synthesis (DNL) and partitioning in response to metformin.

## Subjects and methods

### Study participants

We recruited individuals (six female and four male) who were insulin resistant (*n* = 8) or who were diagnosed with T2D at the time of study enrolment (*n* = 2). All participants were treatment naïve, overweight or obese, and at increased risk of NAFLD. Participants were recruited from the Oxford BioBank (OBB) (www.oxfordbiobank.org.uk) (11) and were aged between 36 and 63 years, had a BMI between 26 and 42 kg/m^2^, were insulin resistant (defined as fasting insulin in the 90th centile of the OBB), and were not taking medications known to alter hepatic metabolism, blood glucose and lipid concentrations, or body weight. Participants were excluded if they consumed excess alcohol (females >14 units/week and males >21 units/week) or had known liver disease. The study was approved by North of Scotland Research Ethics Committee (15/NS/0117) and all subjects gave written informed consent. This physiological study was registered at www.clinicaltrials.org ID number NCT02696941. This included an assessment of sodium-glucose co-transporter 2 treatment on IHTG using similar techniques which have been published elsewhere (12). The focus of the current work was to decipher the precise mechanistic impact of metformin on IHTG and to offer explanations for the inconsistent observations in previous rodent and humans studies.

### Study design

**Figure 1:**
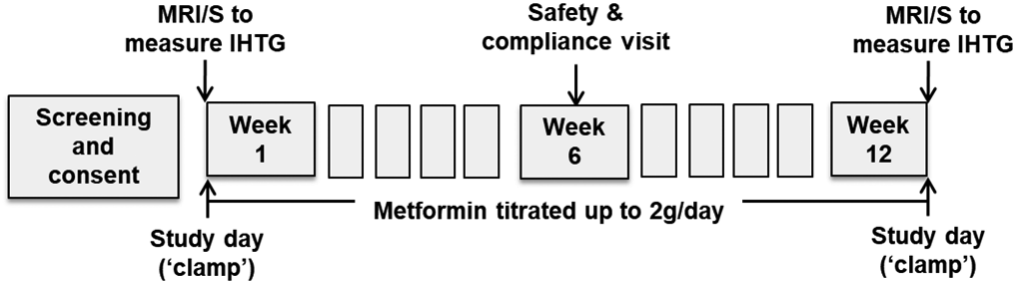
Overview of study design. MRI/S, MRI and spectroscopy; IHTG, intrahepatic triglyceride content; ‘clamp’, two-step hyperinsulinaemic–euglycaemic clamp.

We performed a single centre, open label trial for 12 weeks. Baseline investigations included measurement of IHTG by proton magnetic resonance spectroscopy (^1^H-MRS) (13) and a two-step hyperinsulinaemic–euglycaemic clamp utilising stable-isotope methodologies (12). IHTG content measurements and metabolic assessments were performed prior to and then again after 12 weeks of treatment with metformin, after which all treatment was stopped. Metformin was initiated at 500 mg once a day and increased weekly, as tolerated, by 500 mg up to maximum dose of 1 g twice a day (Fig. 1).

### Metabolic study day

For 48 h prior to the metabolic study days, participants were asked to avoid foods naturally enriched in ^13^C, alcohol, and strenuous exercise. The evening prior to the study day, subjects consumed deuterated water (^2^H_2_O) (3 g/kg body water) and continued to consume ^2^H_2_O during the course of the two-step hyperinsulinaemic–euglycaemic clamp for the measurement of hepatic DNL as described (14). On the study day, participants came to the clinical research unit, where body composition was assessed at the start of the study using impedance.

To assess hepatic and peripheral insulin sensitivity using the two-step hyperinsulinaemic–euglycaemic clamp, arterialised blood was sampled to determine the blood glucose concentration to be maintained (‘clamped’) throughout the study. Steady state blood was sampled at the end of each phase and rates of hepatic endogenous glucose production (EGP) and glucose disposal were calculated using modified versions of the Steele Equations (15). Intravenous infusions of [6,6-D_2_]glucose and [U^13^C]palmitic acid complexed with human albumin (0.03 μmol/kg/min) (to assess the rate of appearance (Ra) of non-esterified fatty acids (NEFA), FA oxidation, and intrahepatic FA partitioning) were commenced at the start of the study day (time 0), and steady state blood samples were taken after 2 h of this basal phase and at two phases on infused insulin as described (12).

Breath samples were collected over the course of each phase in EXETAINER tubes (Labco Ltd, High Wycombe, UK) to determine ^13^CO_2_ production.

### Adipose tissue biopsies

Subcutaneous abdominal adipose tissue samples were taken by needle biopsy from eight participants from the periumbilical area before and after metformin treatment and mRNA was extracted from biopsies, reversed transcribed, and qPCR assays for relevant genes undertaken (16, 17).

### Analytical procedures

Whole blood was collected into heparinized syringes and plasma rapidly separated for the measurement of plasma metabolite and insulin concentrations (18). Separations of VLDL were made by sequential flotation using density gradient ultracentrifugation and expired breath collected as described (18).

### Plasma and breath isotopic enrichment

Gas chromatography-mass spectrometry (GC-MS) was used to determine plasma glucose (19) and FA enrichment (14), and the tracer-to-tracee ratio (TTR) of a sample prior to the administration of the respective tracers was subtracted from the TTR of each sample to account for natural abundance. ^13^CO_2_ production, as a marker of whole-body FA oxidation, was calculated and corrected for lean mass (determined by impedance) (20). Hepatic DNL was based on the incorporation of deuterium from ^2^H_2_O in plasma water into VLDL-TG palmitate using GC-MS (14, 21).

### Plasma metabolomics analysis

We undertook a preliminary assessment of the plasma metabolome before and after metformin treatment. Plasma samples were collected prior to the start of infusions and metabolomics analysis was undertaken using ion chromatography- and reversed-phase-mass spectrometry (MS) methods (22) and for amino acid analysis using liquid chromatography. A 5 μL partial loop injection was used for all analyses and chromatographic separation was performed using a Dionex Ultimate 3000 UHPLC system (Thermo Scientific) coupled to a Q Exactive Hybrid Quadrupole-Orbitrap mass spectrometer (Thermo Scientific). The Q Exactive mass spectrometer was equipped with a heated electrospray ionisation probe in positive ion mode.

### Statistical analysis

We based our power calculations on the work of others, who found a 20–40% decrease in fasting plasma glucose concentrations with metformin treatment between 16 and 96 weeks (23, 24, 25). We recruited individuals who were in the top 20% for fasting glucose concentrations (6.5. ± 0.5 mmol/L (mean ± s.d.)) in the OBB, but without a known diagnosis of T2D. We predicted a conservative decrease of 10% in plasma glucose and with a power of 0.80 at α of 0.05 required 11 individuals. However, as we only recruited ten individuals, who achieved a 5% decrease in plasma glucose concentrations, it is likely the study was underpowered for many of the reported outcomes. Thus, we have used this physiological study as a platform to explore whether metformin affected IHTG content, hepatic DNL, and FA oxidation in humans. Data were analysed using SPSS for Windows v22 (SPSS). Areas under the curve (AUCs) were calculated by the trapezoid method. AUCs have been divided by the relevant time period to give time-averaged values. All data sets were tested for normality according to the Shapiro–Wilk test. Comparisons within the group before and after treatment with metformin were made using a Student’s paired *t*-test or the non-parametric equivalent. Data across the hyperinsulinaemic–euglycaemic clamp were compared using repeated measures ANOVA, with time and treatment as factors to investigate the change. Bonferroni* post hoc* analysis was performed where appropriate to adjust for multiple comparisons. Associations between variables were carried out using Spearman’s rank correlation coefficient. For plasma metabolomics, comparisons were made using MetaboAnalyst (26), and *P* -values were not corrected for multiple testing. For all analysis, statistical significance was set at *P*< 0.05 and unless otherwise stated data are presented as mean ± s.e.m.

## Results

### Anthropometric and fasting biochemical measures

**Figure 2:**
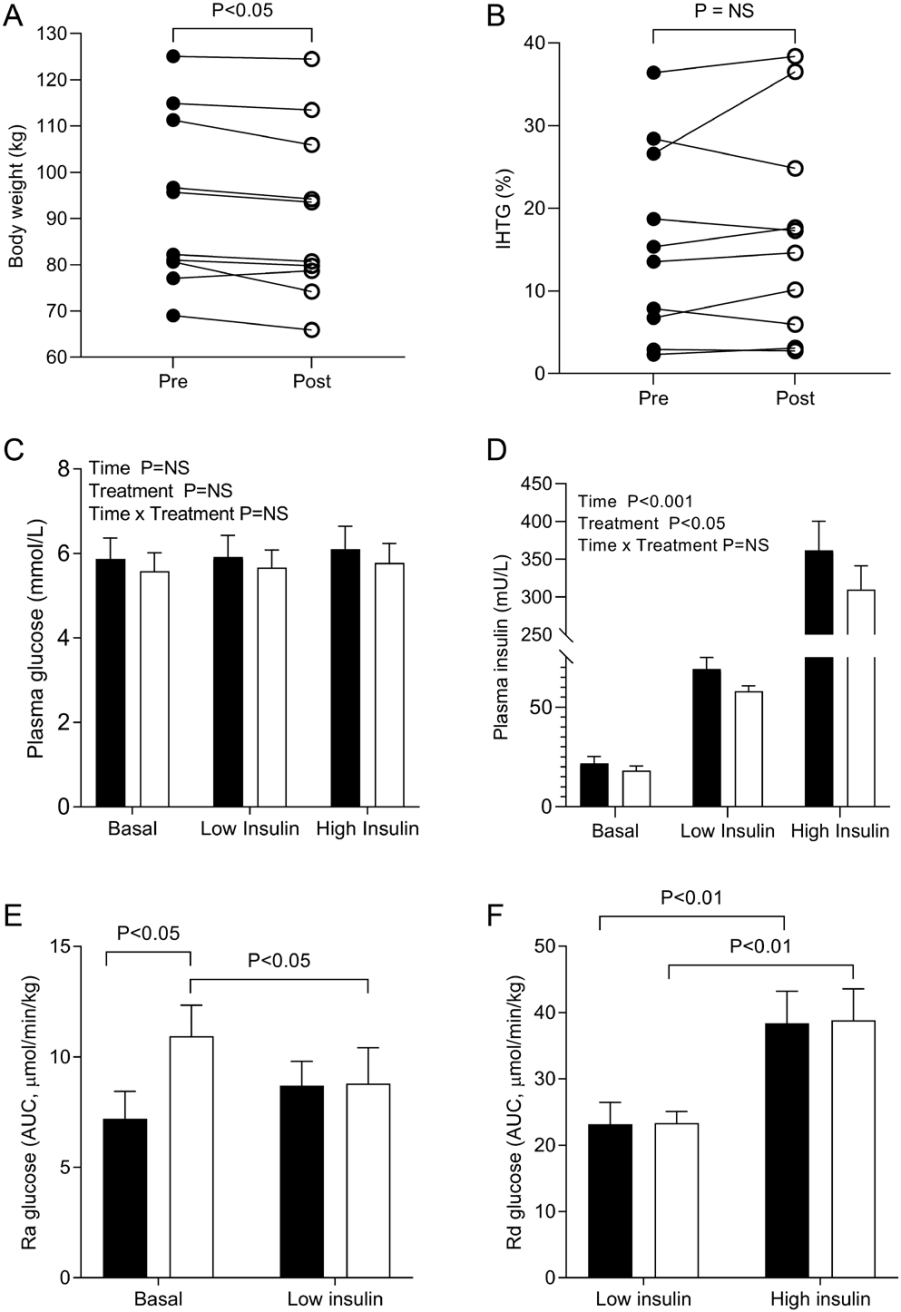
The effect of metformin treatment on (A) body weight (kg); (B) intrahepatic triglyceride (IHTG) %; (C) plasma glucose; (D) plasma insulin; (E) rate of disappearance (Rd) of glucose; (F) rate of appearance (Ra) of glucose at the end of the respective clamp phases during the two-step hyperinsulinaemic–euglycaemic clamp pre (black circles and black bars) and post (open circles and open bars) 12 weeks metformin treatment. Data are reported as mean ± s.e.m.

Ten adults (six females and four males) completed the study (Table 1). After 12 weeks of treatment with metformin, there was a significant (*P*< 0.05) decrease in BMI, which resulted from an average decrease of 2.3 kg in body weight (range: −6.4 to +1.64kg) (Fig. 2A and Table 1), along with non-significant decreases in fat and lean masses; IHTG content remained unchanged (range: −3.6% to +9.9%) (Fig. 2B and Table 1). To ensure that the individual with the largest increase in IHTG was not driving this observation, we excluded them from the analysis and found this had no effect on the outcome. The association between change in body weight and IHTG across the study was not significant (r_s_= 0.58, *P*= 0.08). Plasma lactate significantly (*P*< 0.05) increased and there was no change in plasma total, and HDL- and non-HDL cholesterol concentrations with metformin treatment (Table 1).

**Table 1:** Participant characteristics and plasma biochemical parameters pre and post 12 weeks of treatment with metformin. Data are presented as mean ± s.e.m.

	Pre-metformin	Post-metformin
BMI (kg/m^2^)	33.5 ± 1.3	32.7 ± 1.4^*^
Body weight (kg)	93.4 ± 5.9	91.1 ± 5.9^*^
IHTG (%)	15.9 ± 3.7	17.1 ± 4.0
Fat mass (kg)	34.5 ± 1.9	33.5 ± 2.2
Lean mass (kg)	58.8 ± 1.9	57.6 ± 4.9
HOMA-IR	8.0 ± 2.0	6.3 ± 1.0^*^
Fasting plasma biochemistry		
Glucose (mmol/L)	6.4 ± 0.6	6.1 ± 0.6
Insulin (mU/L)	27 ± 5	23 ± 3
Lactate (mmol/L)	0.82 ± 0.07	1.04 ± 0.08^**^
Total cholesterol (mmol/L)	4.5 ± 0.2	4.4 ± 0.2
HDL cholesterol (mmol/L)	1.1 ± 0.1	1.2 ± 0.2
Non-HDL cholesterol (mmol/L)	3.4 ± 0.3	3.3 ± 0.2
NEFA (µmol/L)	630 ± 66	604 ± 53
Triglycerides (mmol/L)	1.7 ± 0.4	1.4 ± 0.3^†^
VLDL-TG (µmol/L)	954 ± 181	726 ± 194^*^
VLDL-ApoB (µmol/L)	0.09 ± 0.02	0.07 ± 0.01
VLDL-TG/VLDL-ApoB molar ratio	8833 ± 770	8300 ± 1068
3-hydroxybutyrate (µmol/L)	91 ± 18	88 ± 13

^†^*P*= 0.06, ^*^P< 0.05, ^**^P< 0.01 pre vs post 12 weeks of metformin treatment.

ApoB, apolipoprotein B; IHTG, intrahepatic triglyceride; NEFA, non-esterified fatty acids; TG, triglyceride.

### Insulin sensitivity plasma glucose and insulin

The Homeostasis Model Assessment for Insulin Resistance (HOMA-IR) significantly (*P*< 0.05) decreased after 12 weeks treatment with metformin (Table 1); there were non-significant decreases in plasma glucose and insulin concentrations (Table 1). Over the course of the clamp, there was no change in plasma glucose concentrations (Fig. 2C), although the amount of insulin required to maintain glucose homeostasis was significantly (*P*< 0.05) lower in both the low and high insulin phases after metformin treatment (Fig. 2D). The decreased insulin may be due to metformin treatment leading to an increase in insulin clearance and/or is a reflection of the decrease in body weight, as infusion rates were calculated on a per body mass (kg) basis. There was a non-significant increase in the rate of disposal (Rd) of glucose after metformin treatment (9.7 ± 1.0 (pre) vs 12.3 ± 1.9 (post) µmol/min/kg) in the basal state. Rd glucose then significantly (*P*< 0.01) increased between the low and high insulin clamp phases with no effect of treatment (Fig. 2E). In the basal phase, Ra glucose increased significantly (*P*< 0.05) post-metformin when compared to pre-metformin treatment (Fig. 2F). Between the basal and low insulin phase, Ra glucose significantly decreased post-metformin test but not pre-metformin treatment (Fig. 2F).

### Adipose tissue FA metabolism

**Figure 3:**
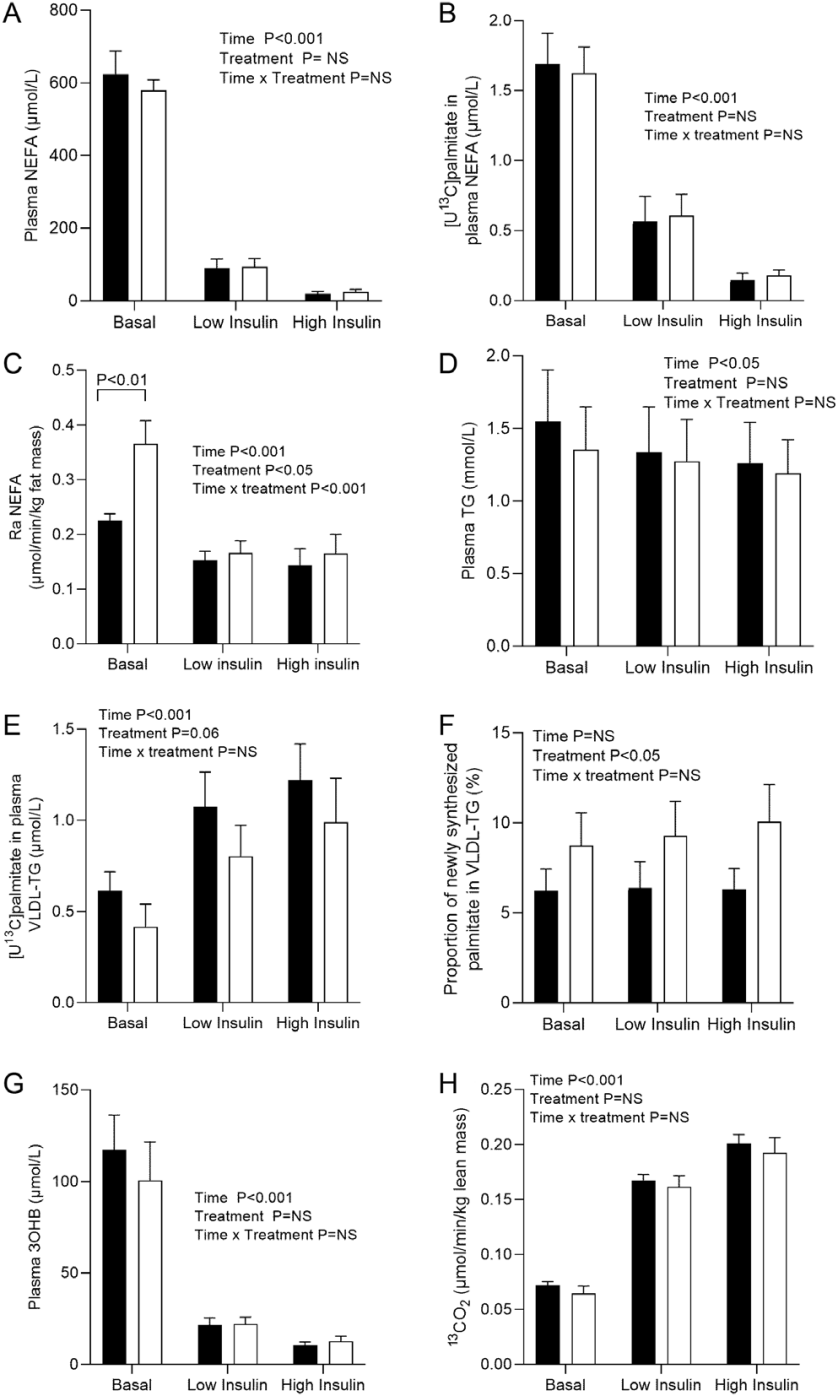
The effect of metformin treatment on (A) plasma non-esterified fatty acids (NEFA); (B) the incorporation of [U^13^C]palmitate in plasma NEFA; (C) the rate of appearance (Ra) of plasma (NEFA), (D) plasma triglycerides (TG); (E) the incorporation of [^13^C]palmitate in VLDL-TG; (F) proportion of newly synthesised palmitate in VLDL-TG (hepatic *de novo* lipogenesis); (G) plasma 3-hydroxybutyrate (3OHB); and (H) ^13^CO_2_ production at the end of the respective clamp phases during the two-step hyperinsulinaemic–euglycaemic clamp pre ( black bars) and post (open bars) 12 weeks metformin treatment. Data are reported as mean ± s.e.m.

**Figure 4:**
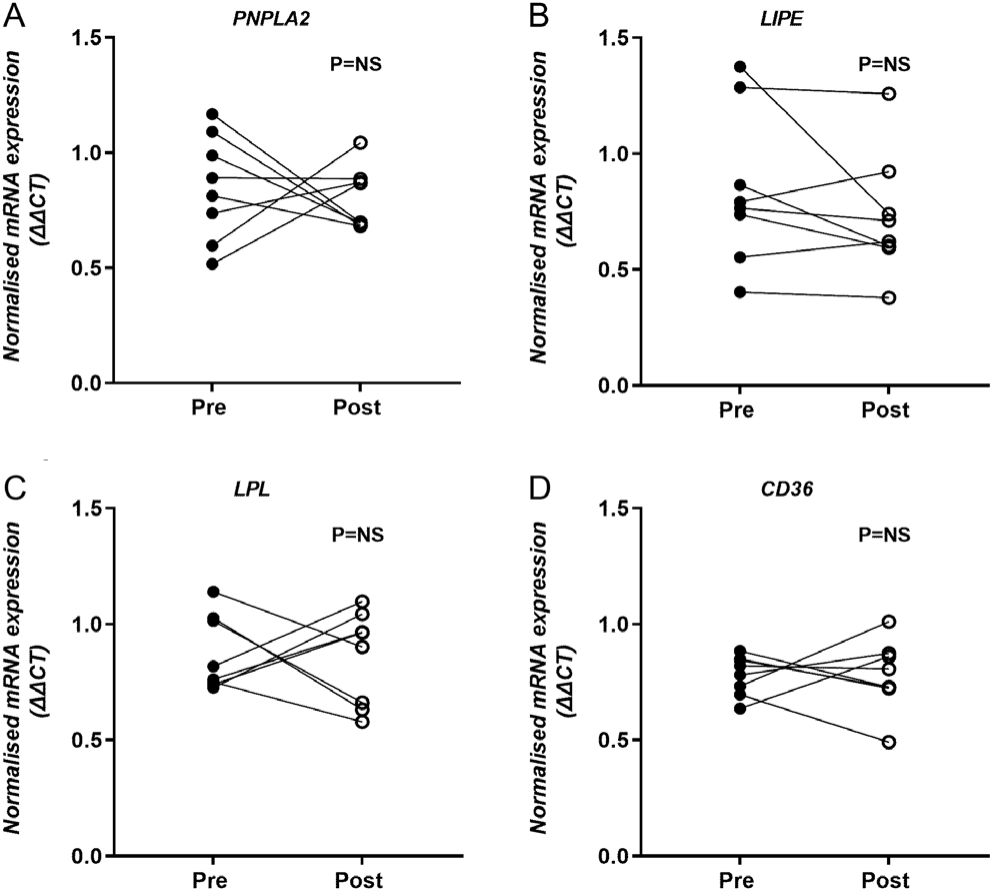
Change in the s.c. abdominal adipose tissue expression of (A) adipose triglyceride lipase (*PNPLA2*); (B) hormone-sensitive lipase (*LIPE*); (C) lipoprotein lipase (*LPL*); and (D) fatty acid translocase (*CD36*) pre (black circles) and post (open circles) 12 weeks metformin treatment.

Metformin treatment had little effect on plasma NEFA concentrations either in the basal state or during the study day, with the expected suppression occurring over the course of the insulin clamp (Fig. 3A). By including an i.v. infusion of [U^13^C]palmitate complexed with human albumin, we were able to assess the effect of metformin treatment on s.c. adipose FA trafficking. We observed no difference in the incorporation of [U^13^C]palmitate into the plasma NEFA pool over the course of the clamp between the pre- and post-metformin measurements (Fig. 3B). Despite no difference in NEFA concentrations, we unexpectedly observed a significant increase in RaNEFA post- compared to pre-metformin treatment (time × treatment *P*< 0.001), which appeared to be largely driven by a significant (*P*< 0.01) increase during the basal period post-metformin treatment (Fig. 3C). As the increase in RaNEFA may have been due to a change in insulin sensitivity, we assessed the correlation with plasma insulin concentrations and found no association (r_s_= −0.02, *P*= 0.68) pre-metformin but a significant inverse association (r_s_= −0.71, *P*= 0.022) post-metformin. We examined the correlation between pre- and post-metformin for RaNEFA and IHTG and found a tendency for an inverse association (r_s_= −0.61, *P*= 0.06) but no correlation between RaNEFA and body weight (r_s_= −0.29, *P*= 0.43). To determine if the increase in RaNEFA was due to changes in adipose tissue FA trafficking and metabolism, we assessed the expression of relevant genes in s.c. abdominal adipose tissue biopsies taken pre- and post-metformin treatment. Although the expression of genes involved in FA trafficking remained unchanged (Fig. 4A,B, C and D), interlukin 6 (*IL6*) expression significantly (*P*< 0.05) decreased (from 1.24 ± 0.17 to 0.86 ± 0.05 normalised mRNA expression ΔΔCT) with metformin treatment.

### Plasma and VLDL-TG and hepatic DNL

Although there was a tendency (*P*= 0.06) for fasting plasma TG concentrations to decrease after metformin treatment (Table 1), we did not observe a treatment effect over the course of the insulin clamp (Fig. 3D). Fasting plasma VLDL-TG significantly decreased (*P*< 0.05) with metformin treatment (Table 1), with no further differences observed over the course of the insulin clamp (data not shown). Although the VLDL-TG/VLDL-ApoB molar ratio in the fasting state (Table 1) and over the course of the insulin clamp (low insulin: 8971 ± 1104 vs 8156 ± 1207 and high insulin: 8184 ± 995 and 7009 ± 952 pre- vs post-metformin, respectively) was lower post-metformin treatment compared to pre-metformin treatment, the changes were not significant.

Systemic NEFA can be taken up by the liver and utilised for the synthesis of TG which can then either be secreted in VLDL or stored in cytosolic lipid droplets. We assessed the appearance of [U^13^C]palmitate (from the [U^13^C]palmitate infusion) into VLDL-TG and found the increase over time tended (*P*= 0.06) to be lower with metformin treatment (Fig. 3E); we did not measure VLDL-TG production or clearance rates. Metformin treatment had no effect on either the relative or absolute contribution of systemic NEFA to VLDL-TG (data not shown). Over the course of the insulin clamp, metformin treatment significantly (*P*< 0.05) increased the relative contribution of DNL FA to VLDL-TG (Fig. 3F); however, when expressed in absolute terms, this effect disappeared (data not shown). We found the relative change in hepatic DNL was positively associated with change in IHTG content (r_s_= 0.75, *P*< 0.05) while the absolute change was not (r_s_= 0.52, *P*= 0.13).

### Hepatic ketogenesis and whole-body FA oxidation

Metformin treatment had no effect on plasma 3-hydroxybutyrate (3OHB) concentrations either in the fasting state or over the course of the clamp; 3OHB concentrations were suppressed to nadir by the end of the low insulin phase (Fig. 3G). We measured the appearance of ^13^C (from [U^13^C]palmitate) into expired CO_2_ as a marker of whole-body FA oxidation and found no effect of metformin treatment on ^13^CO_2_ production in the basal, low, or high insulin phases (Fig. 3H).

### Plasma metabolomics

**Figure 5:**
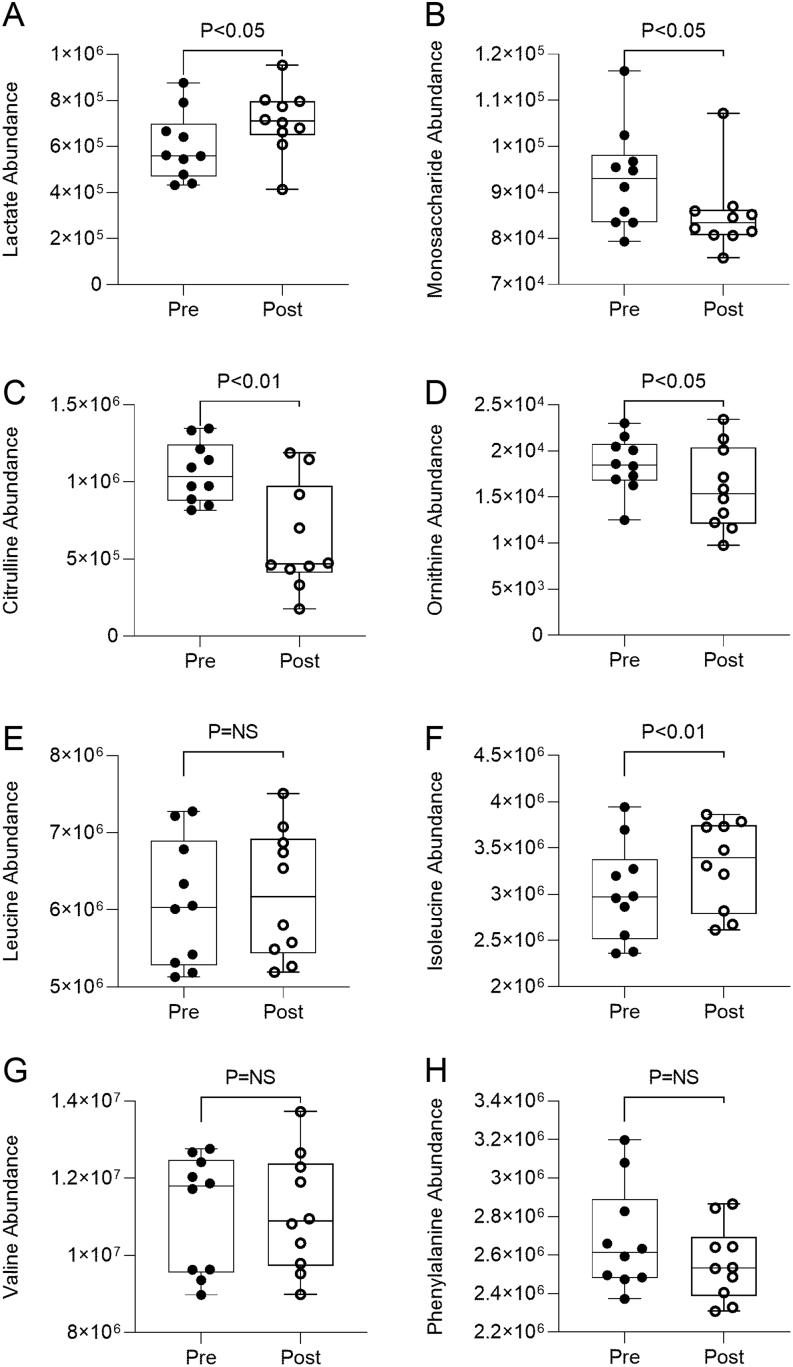
The plasma metabolome and the abundance in plasma (A) lactate, (B) monosaccharide, (C) citrulline, (D) ornithine, (E) leucine, (F) isoleucine, (G) valine, and (H) phenylalanine pre (black circles) and post (open circles) 12 weeks metformin treatment. Data are reported as median ± range.

We had the opportunity to investigate if changes occurred in the plasma metabolome with metformin treatment for 12 weeks. In our preliminary analysis, relatively few changes were identified. In-line with the plasma biochemical data, plasma lactate (IC FC1.19 p0.0199) levels significantly increased (~one-fold higher, *P*< 0.05) after metformin treatment (Fig. 5A). There were significant (*P*< 0.05) decreases in the plasma levels of total monosaccharide (which is not only composed predominantly of glucose but also includes fructose, galactose, and mannose) and the amino acids citrulline (AAA FC1.69 p0.0021) and ornithine (AAA FC1.16 p0.0184) in plasma post- compared to pre- metformin treatment (Fig. 5B, C and D). Of the three branched chain amino acids, only isoleucine (AAA FC1.10 p0.0012) was significantly (*P*< 0.01) higher post-metformin treatment (Fig. 5E and F); there was no change in the level of the aromatic amino acid phenylalanine with metformin treatment (Fig. 5G).

## Discussion

The present study was undertaken to examine the paradoxical observations of metformin treatment improving insulin sensitivity and plasma lipid profiles despite having inconsistent effects on IHTG content. Overall, we found that 12 weeks of metformin treatment resulted in a significant decrease in body weight and markers of insulin resistance. Despite these beneficial changes, we observed no effect on IHTG content or whole-body FA oxidation and a significant increase in the relative contribution of DNL FA to VLDL-TG. Our findings go some way towards explaining some of the observed clinical disconnect with metformin treatment, between the metabolic benefits of weight loss, yet no impact on IHTG.

The most consistent finding from studies in adults is that metformin treatment decreases body weight (27); however, the effect on IHTG is less clear (8). In the present study, body weight decreased by 2.5% with metformin treatment, with no change in IHTG content, which is in-line with previous studies that have utilised ^1^H-MRS to quantify IHTG (25, 28). It is plausible that the decrease in body weight was not sufficient to induce a change, although others have reported IHTG content to decrease with weight loss between 1.1 and 8.0% after metformin treatment (8). The discrepancy in findings may, in part, be explained by the methodology utilised to assess IHTG. Despite the lack of change in IHTG, we and others (27) found metformin treatment to decrease markers of insulin resistance in individuals with and without T2D. In contrast to previous observations in non-diabetic participants (29, 30, 31), we did not observe a significant increase in Rd glucose. However, we observed a significant increase in the basal state Ra glucose after metformin treatment, which suggests an increase in EGP. Others have reported EGP to increase in non-diabetic participants after metformin treatment (32); it has been suggested that the mechanism of action of metformin may be dependent on the glycaemic state of the individual (30, 31). Moreover, it is becoming more evident that the mechanism of action of metformin maybe extra-hepatic; it has been speculated that changes in glucose clearance after metformin treatment could represent increased intestinal glucose uptake (31). We also observed a significant decrease in the amount of insulin required to maintain glucose homeostasis after metformin treatment, which may be the result of metformin increasing insulin clearance, as reported by some (33) but not others (25).

Treatment with metformin has been suggested to increase hepatic glucose uptake in humans (34), which may then be partitioned into glycogen storage, oxidised, or utilised in the DNL pathway. We observed a significant increase in the relative contribution of DNL FA to VLDL-TG after 12 weeks treatment with metformin. The change in the relative contribution of DNL FAs between pre- and post-metformin treatment was positively associated with the change in IHTG content, suggesting DNL played a role in maintenance of IHTG. It is also plausible IHTG was maintained due to decreased TG export in VLDL. We observed a significant decrease in VLDL-TG concentrations following metformin treatment, along with a non-significant decreases in VLDL-ApoB concentrations (reflecting particle number) and the VLDL-TG/VLDL-ApoB molar ratio (reflecting particle lipidation). These changes may be due to a (subtle) decrease in VLDL secretion, which would contribute to maintenance of IHTG content. Gormsen *et al.* (35) reported metformin treatment had no effect on VLDL-TG concentrations, the TG/ApoB ratio, or VLDL-TG Ra. It remains unclear if metformin treatment affects the rate of clearance of VLDL-TG in humans. In-line with Gormsen *et al.* (35), we observed no change in whole-body FA oxidation or plasma 3OHB concentrations with metformin treatment. The lack of change in FA oxidation and ketogenesis, in combination with the increase in hepatic DNL may, in part, explain the maintenance of IHTG content, as cellular conditions would be favouring FA esterification (36).

Elevated plasma NEFA are often suggested as underlying cause of NAFLD/insulin resistance. Although we found a significant time and treatment effect of metformin on RaNEFA, in agreement with others (34, 35) we found no notable change in systemic NEFA concentrations. Despite the higher basal RaNEFA, following metformin treatment, maximal suppression was achieved at the low insulin dose. Gormsen *et al.* (35) have previously reported that metformin treatment had no effect on whole-body FA turnover or hepatic FA uptake, while others have reported metformin treatment to increase plasma NEFA concentrations in patients with T2D but not control participants (30). Based on the significant inverse association we observed between plasma insulin and RaNEFA, it is plausible that adipose tissue became more insulin sensitive after metformin treatment. We have previously found RaNEFA to be higher (per unit of fat mass) in lean insulin-sensitive compared to overweight/obese insulin-resistant males (16). Karpe *et al.* (37) also reported that lower plasma insulin levels were associated with a higher NEFA release (per unit of fat mass) from s.c. abdominal adipose tissue. As we have previously found the expression of FA trafficking genes to be significantly lower in obese, insulin-resistant compared to lean, insulin-sensitive s.c. adipose tissue (16), we explored whether metformin affected relevant pathways and found no effect on the expression of FA trafficking genes. Others have reported 6 weeks of metformin treatment in older (71 ± 6.4 years), overweight (29 ± 3.6 kg/m^2^) adults (*n* = 14) influenced metabolic genes involved in FA pathways in s.c. abdominal adipose tissue (38). As RdNEFA is assumed to equal RaNEFA (39), we cannot exclude the increase in RaNEFA we observed was due to decreased NEFA clearance rather than increased intracellular TG lipolysis.

We found a significant decrease in the expression of adipose tissue *IL6* after metformin treatment. Although we did not measure plasma IL6 concentrations, it would be of interest to determine the effects of metformin on adipose tissue macrophage polarisation as this may influence adipose tissue inflammation. Adeshara *et al.* (40) found that 3 months of treatment with metformin in individuals with T2D significantly decreased plasma IL6 concentrations. We have previously found the release of IL6 to be significantly lower from gluteofemoral compared to abdominal s.c. adipose tissue (41), suggesting changes in plasma IL6 concentrations will be driven by changes in abdominal s.c. adipose tissue metabolism.

In-line with others (42, 43), we observed notable decreases in the plasma levels of citrulline and ornithine following metformin treatment. Both amino acids have prominent roles in the urea cycle and nitric oxide biosynthesis (42). It has been suggested that a metformin-induced decrease in these metabolites may reflect reductions in urea biosynthesis, secondary to reductions in gluconeogenesis (44), although the mechanism(s) responsible remain unclear. Elevated levels of branch chain and aromatic amino acids have been positively associated with T2D risk (45). Although we observed an increase in isoleucine levels after metformin treatment, the relevance remains unclear as there was no effect on leucine or valine levels. Gormsen *et al.* (30) undertook non-targeted metabolomics analysis on plasma collected from patients with T2D and control subjects before and during 3 months of treatment with metformin. They found changes in amino acid, carbohydrate, and FA pathways and commented that the effect of metformin on the plasma metabolome was dependent on the presence of overt diabetes (30). The effects of an acute, single-dose of metformin on the plasma metabolome has been investigated. Rittig *et al.* (46) reported that 90 min after patients with cirrhosis had taken 1000 mg of metformin, tricarboxylic acid (TCA) cycle intermediates were elevated suggesting increased tissue glycolysis, along with decreased microbiome-derived metabolites linked to benzoate and hippurate metabolism. Others have found significant changes in pathways linked with valine, leucine and isoleucine biosynthesis, phenylalanine metabolism, and FA metabolism after healthy males were given 500 mg of metformin (47). Thus, it is plausible that the lack of change in the plasma metabolome observed in our study was due to participants not having overt T2D and they had not consumed metformin within hours of the measurement.

Our study is not without limitations. We studied a small, heterogeneous cohort of individuals with and without T2D, and this most likely explains the variability in response we observed with metformin treatment. Given our small sample size, it is likely our study was underpowered for many of the outcomes measured. As we did not have a placebo or comparator group, we are limited in drawing firm conclusions about whether the effects we have observed here were due solely to metformin treatment. However, despite the heterogeneity within the group, we observed a robust increase in the relative contribution of DNL FA to VLDL-TG and a clear lack of change in whole-body FA oxidation. We used impedance to assess body composition which is reliant on total body water status, and it is therefore possible we have under or overestimated the changes in fat and lean masses. Although we asked participants to maintain their habitual lifestyle and dietary habits, we did not assess or control dietary intakes and therefore it is plausible that the upregulation in hepatic DNL is the result of an increased intake of carbohydrate.

There is clear evidence for the clinical benefits of metformin treatment in individuals with T2D and insulin resistance as it notably improves insulin sensitivity and may lower the risk of CVD which is potentially mediated through improved lipid profiles (27), despite having little effect on IHTG content. Although our data suggest that metformin may not have a direct impact on IHTG content and would support existing recommendations that NAFLD alone is not an indication for initiation of treatment (48), this must be carefully balanced against the wider, well-established benefits of metformin. Here we have presented the effect of metformin on multiple processes that govern IHTG accumulation and may help explain some of the variabilities in clinical data that have previously been reported. The data from this study have unravelled the disconnect in the mechanisms of action of metformin whereby it coveys metabolic benefits and weight loss, without improvement in IHTG; these observations are explained, at least in part, through increased hepatic DNL.

## Declaration of interest

The authors declare that there is no conflict of interest that could be perceived as prejudicing the impartiality of this study.

## Funding

The study was funded by a Novo Nordisk http://dx.doi.org/10.13039/501100004191 Postdoctoral Fellowship run in partnership with the University of Oxford (C J G) and a British Heart Foundationhttp://dx.doi.org/10.13039/501100000274 Senior Research Fellowship in Basic Science (FS/15/56/31645 awarded to L H).
